# Primary Care and Linked Secondary Care Encounters for Foot and Ankle Problems in Children and Young People: A Population‐Based Cohort Study in England

**DOI:** 10.1002/jfa2.70046

**Published:** 2025-04-15

**Authors:** Emma Rezel‐Potts, Catherine Bowen, Kate M. Dunn, Christopher I. Jones, Martin C. Gulliford, Stewart C. Morrison

**Affiliations:** ^1^ School of Life Course and Population Sciences King's College London London UK; ^2^ Faculty of Environmental and Life Sciences University of Southampton Southampton UK; ^3^ Centre for Musculoskeletal Health Research School of Medicine Keele University Keele UK; ^4^ Department of Primary Care and Public Health Brighton and Sussex Medical School Falmer UK

**Keywords:** adolescent, ankle, child, foot, general practitioners, hospitals, secondary care

## Abstract

**Background:**

In the United Kingdom, foot and ankle problems in children and young people are typically seen by the general practitioner in primary care and referred to secondary care or community services for specialist assessment and intervention. Following initial presentation to primary care, we have described the secondary care services accessed by children and young people with foot and ankle problems. We have also explored the sociodemographic variables associated with referrals to secondary care.

**Method:**

This was a population‐based cohort study using the UK Clinical Practice Research Datalink (CPRD) Aurum primary care database and linked Hospital Episode Statistics (HES) Outpatient database. We extracted data for all children and young people up to 18 years of age with a consultation for a foot and ankle problem from 1st January 2015 to 31st December 2021 (CPRD) and included those with linked data in secondary care (HES database) in our analyses.

**Results:**

346,454 children and young people aged 0–18 years were identified in CPRD and eligible for linkage; 5030 had at least one referral within 18 weeks. The most common reason for referral was musculoskeletal or unspecified pain and 2935 had a referral to trauma and orthopaedics, 1314 for paediatric services, 678 for physiotherapy and 274 for diagnostic imaging. Odds for referrals were higher among younger age groups (odds ratio (OR) 1.29 and 95% confidence interval (CI) 1.25–1.33). Those in other (OR 0.77 and 95%CI 0.72–0.82), Asian (OR 0.81 and 95%CI 0.77–0.86) and Black (OR 0.85 and 95%CI 0.8–0.91) ethnic groups had lower odds of referral compared to those in the White group.

**Conclusion:**

These findings represent the first analyses of secondary care referrals for children and young people with foot and ankle problems. We have identified that musculoskeletal symptoms were most common reason for referral and the most common speciality involved in assessing foot and ankle problems was trauma and orthopaedics. We have reported sociodemographic differences in secondary care referrals and these findings could be indicative of inequalities in access to care and should be a priority for further research.

**Abbreviations**
CPRD: Clinical Practice Research Datalink
CYP: children and young People
ED: emergency department
HES OP: Hospital Episode Statistics Outpatient
ICD‐10: International Statistical Classification of Diseases and Related Health Problems
IMD: Index of Multiple Deprivation
NEC: Not elsewhere classifiable

## 1 Introduction

Unintentional injuries, dermatological issues, musculoskeletal disorders and other noncommunicable diseases are common causes of morbidity among children and young people in England, UK [1]. We previously identified that foot and ankle problems are typically linked to these causes [2] and access to primary care services are important to enable referral on to secondary healthcare services. However, the National Health Service (NHS) in England, UK is struggling to shift to a model of integrated patient‐centred care, across primary and specialist services, which is needed to effectively address these problems. Challenges to the delivery of services include system fragmentation, workforce shortages, budget constraints and rising costs [3, 4, 5]. It has been documented that children and young people are increasingly attending secondary care services for urgent care and outpatient visits (a planned appointment with a hospital specialist) [6], and further work is needed to understand the secondary care services involved in managing foot and ankle problems.

Foot and ankle problems can have serious impact on a range of outcomes affecting children and young people, including development [7], school attendance and engagement [8], self‐consciousness and abilities to participate in life‐events alongside their peers [8, 9, 10, 11, 12]. These can also impact longer‐term health, well‐being and social mobility [7, 8, 13] and have negative effects on parents and caregivers [14]. Despite the importance for children and young people, and their potential as indicators of effective health system performance, foot and ankle health in children and young people is a neglected area of research. Our analysis of English primary care data from 2015 to 2021 found that the average rate of foot and ankle consultations was 343 per 10,000 patient years [2]. Many of these consultations were for causes categorised as musculoskeletal, dermatological and unspecified pain, all of which would likely warrant onward referral to specialists for accurate diagnosis and treatment [2]. However, very little is known about the management of these problems through the healthcare system. Building upon this previous work, we describe the secondary care services accessed by children and young people with foot and ankle problems who were initially seen in the primary healthcare setting. We also explore the sociodemographic variables associated with referrals to secondary care.

## 2 Methods

### 2.1 Study Population and Data Sources

This population‐based cohort study employed the UK Clinical Practice Research Datalink (CPRD) Aurum, a database of electronic health records for UK primary care and the Hospital Episode Statistics Outpatient (HES OP), a database of secondary care outpatient appointments occurring in England. Each patient in the CPRD has a unique anonymised numerical identifier that remains the same at each update and which can be individually linked to secondary care and area‐based datasets including HES OP. The CPRD Aurum May 2022 release has a total of over 41 million acceptable patients, covering approximately 20% of the population of England [15], with good representativeness in terms of geographical distribution, deprivation, age and gender [16]. Among these acceptable patients, 93% are eligible for linkage [15].

The CPRD includes coded recording of prescriptions and clinical diagnoses from general practice. HES OP includes information on the type of outpatient consultation appointment dates, the main speciality and treatment speciality under which the patient was treated and referral source. HES OP has been established as valid for research purposes [17]. Further linked socioeconomic data were obtained from the Index of Multiple Deprivation (IMD) for patient postcode and practice postcode. The study was reviewed for ethical and methods content via Research Data Governance Application and approved by the CPRD team (electronic research application portal protocol number 20_ 002,137). All work was conducted in accordance with the Declaration of Helsinki.

We extracted data for all children and young people up to the age of 18 years with a consultation for foot and/or ankle problem during the period 1st January 2015 to 31st December 2021 in CPRD Aurum based on previously derived codelists [18]. We included only those who were eligible for linkage and with the latest general practice consultation for foot and ankle problems before 27th June 2020 to enable a minimum of 18 weeks follow‐up (maximum waiting time for nonurgent consultant‐led treatment [19] in lieu of equivalent standards for allied health professionals).

The main study outcome was an appointment in HES OP [20] within 18 weeks of any consultation for foot and ankle problems in the CPRD for treatment specialities. Within the United Kingdom, 18 weeks is the expected maximum waiting time for nonurgent consultant‐led treatment [19] and was deemed a reasonable limit for our analyses. Based on the collective experience of the research team, the treatment specialities that we deemed relevant to foot and ankle referrals were: paediatric service; trauma and orthopaedic service; physiotherapy service; paediatric trauma and orthopaedic service; dermatology service; community paediatric service; diagnostic imaging service; paediatric dermatology service; podiatric surgery service; podiatry service and sport and exercise medicine service. We also described overall HES OP attendance within 18 weeks of any consultation for foot and ankle problems in the CPRD according to treatment speciality, main speciality, source of referral, attendance and the presence of any foot and ankle primary or secondary diagnosis using a list of ICD‐10 codes. It is not mandatory to record diagnostic information in HES OP and is understood to be available for less than 5% of all attendances.

Covariates were defined using data recorded in the study period before the index date. Covariates were selected because of known associations with the foot and/or ankle and were identified using the primary care records. These were age category (0–4 years, 5–9 years, 10–14 years and 15–18 years), gender (male or female—covariate and category terminology as specified by CPRD), region of practice, typical pre‐existing health conditions, which impact the foot and ankle (autism, lupus, juvenile arthritis, intellectual disability, diabetes, cerebral palsy and attention‐deficit hyperactivity disorder) and body mass index (BMI). The BMI values were converted to Z‐scores and adjusted for age and gender using the British 1990 growth reference data population [21]. Normal weight was defined as a BMI Z‐score < 1.04 (< 85th percentile on a growth chart). Overweight was defined as 1.04 to 1.64 (85th to 95th percentile) and obese as a Z‐score of ≥ 1.64 (≥ 95th percentile) of the United Kingdom 1990 reference population [21]. Ethnicity data were obtained from HES and were classified as ‘white,’ ‘black,’ ‘Asian’, ‘mixed,’ ‘other,’ and ‘not known’. Linked social deprivation data were derived from the participant postal code of residence and the practice postal code based on IMD 2019 classification at lower super output area, divided into quintiles based on the national distribution from first quintile (most deprived) to fifth quintile (least deprived). The IMD is derived from seven domains of deprivation (income, employment, education, health, crime, housing and quality of living environment).

### 2.2 Analysis

Descriptive characteristics were ascertained for the total number of children and young people accessing primary care for foot and ankle problems who were eligible for linkage. We used hierarchical multivariable logistic regression analysis to evaluate sociodemographic associations and existing health conditions with HES OP appointment for relevant treatment specialities within 18 weeks of any consultation for foot and ankle problems in the CPRD. Included in the model were gender, age category, ethnic group and practice IMD with practice identifier as random effects. Analyses were performed using R version 4.2.3 [22]. The ‘stats’ package [23] was used for analysis, and ‘ggplot2’ [24] and ‘forestplot’ [25] were used to construct plots.

## 3 Results

### 3.1 Characteristics of Study Population

There were 346,454 patients with primary care encounters for foot and ankle problems from 1st January 2015 to 27th July 2020 from 1441 practices who were also eligible for linkage to HES data. Among these, 5030 (1%) had at least one referral to relevant treatment specialities according to HES OP data. Descriptive characteristics for the cohort are presented in Table 1. The mean age of the study population was 10.7 years (standard deviation, 4.6) and the age category with the highest frequency of patients was 10–14 years for both those with no referrals (42%) and those with referrals (43%). Among those with no referrals, the vast majority had only one primary care encounter for foot and ankle during the study period (66%), whereas those with referrals had one primary care encounter (33%) and many with two (24%) or three to five encounters (30%). Participants were mostly in the White ethnic group (no relevant referrals: 77% and relevant referrals 84%), followed by Asian, Black and ‘Other’. Practices were mostly in the least deprived quintile of deprivation (no relevant referrals: 24% and relevant referrals 25%) according to their postcode IMD and were most likely to be situated in the Southeast and Northwest regions of England. Among those with relevant referrals for foot and ankle problems, autism was the most common of the conditions (6%), followed by attention deficit hyperactivity disorder (ADHD) (4%) and intellectual disability (4%).

**TABLE 1:** Cohort characteristics and outcome frequencies.

		Patients with no relevant^a^ secondary care referral	Patients with relevant^a^ HES OP appointment within 18 weeks of any consultation for foot and ankle health in the CPRD
Total		341,424	5030
No. encounters in primary care			
	One	225,986 (66)	1671 (33)
	Two	65,451 (19)	1197 (24)
	Three to five	42,002 (12)	1492 (30)
	Six to ten	6908 (2)	508 (10)
	More than ten	1077 (0)	162 (3)
Foot and ankle problem^a^			
	Musculoskeletal	128,248 (38)	1986 (39)
	Unspecified pain	78,762 (23)	969 (19)
	Dermatological	58,326 (17)	628 (12)
	Infection	36,080 (11)	401 (8)
	Fracture	24,943 (7)	763 (15)
	Miscellaneous	13,677 (4)	220 (4)
	Surgical procedure	1174 (0)	55 (1)
	Nerve	175 (0)	8 (0)
	Tumour	29 (0)	0 (0)
	Circulatory issue	10 (0)	0 (0)
Age group (years)			
	0 to 4	43,596 (13)	842 (17)
	5 to 9	76,703 (22)	1257 (25)
	10 to 14	142,578 (42)	2150 (43)
	15 to 18	78,547 (23)	781 (16)
Gender			
	Male	176,920 (52)	2650 (53)
	Female	164,504 (48)	2380 (47)
Body mass index (z)^b^			
	Normal weight	21,686 (6)	409 (8)
	Overweight	6998 (2)	138 (3)
	Obese	5010 (1)	135 (3)
	Unknown	307,730 (90)	4348 (86)
Ethnic group			
	White	263,560 (77)	4201 (84)
	Asian	24,130 (7)	296 (6)
	Black	14,845 (4)	173 (3)
	Mixed	12,313 (4)	141 (3)
	Other	11,800 (3)	180 (4)
	Not known	14,776 (4)	39 (1)
Index of multiple deprivation (practice)			
	First quintile (most deprived)	60,270 (18)	821 (16)
	Second quintile	57,710 (17)	878 (17)
	Third quintile	70,044 (21)	990 (20)
	Fourth quintile	72,745 (21)	1077 (21)
	Fifth quintile (least deprived)	80,655 (24)	1264 (25)
Index of multiple deprivation (patient)			
	First quintile (most deprived)	73,260 (21)	1060 (21)
	Second quintile	66,107 (19)	930 (18)
	Third quintile	63,056 (18)	854 (17)
	Fourth quintile	65,968 (19)	1019 (20)
	Fifth quintile (least deprived)	72,730 (21)	1164 (23)
	Unknown	303 (0)	3 (0)
Region			
	South East	72,705 (21)	1106 (22)
	North West	64,430 (19)	1102 (22)
	South West	43,218 (13)	674 (13)
	West Midlands	56,247 (16)	848 (17)
	London	59,085 (17)	705 (14)
	East of England	17,029 (5)	167 (3)
	North East	11,856 (3)	209 (4)
	Yorkshire and the Humber	11,107 (3)	120 (2)
	East Midlands	5747 (2)	69 (1)
Pre‐existing health conditions			
	Lupus	84 (0)	6 (0)
	Juvenile arthritis	616 (0)	44 (1)
	Intellectual disability	6407 (2)	186 (4)
	Diabetes	5689 (2)	91 (2)
	Autism	11,996 (4)	309 (6)
	Cerebral palsy	1005 (0)	60 (1)
	ADHD	9207 (3)	217 (4)

*Note:* Figures are frequencies (column percent) except where indicated.

^a^ Specialities: paediatric service; trauma and orthopaedic Service; physiotherapy service; paediatric trauma and orthopaedic service; dermatology service; community paediatric service; diagnostic imaging service; paediatric dermatology service; podiatric surgery service; podiatry service and sport and exercise medicine Service.

^b^ Z scores using BMI measures in the year prior or after the index date or date of any consultation and not including measures recorded at ages below 3 years.

## 4 Secondary Care Treatment Specialities

**FIGURE 1:**
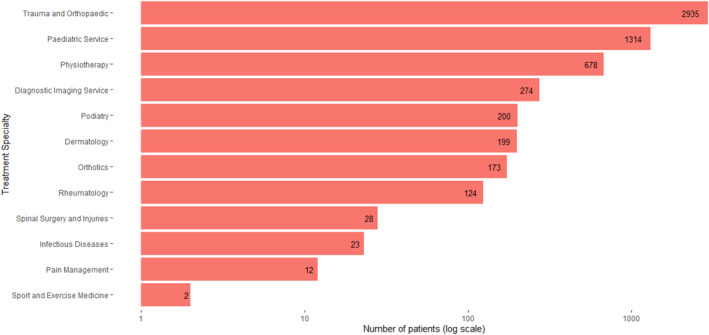
Number of patients referred to treatment specialities in Hospital Episode Statistics Outpatient records within 18 weeks of any primary care appointment from 1st January 2015 to 27th June 2020. Individual patients may have referrals for more than one speciality.

Figure 1 reports the number of patients with appointments for treatment specialities in HES OP within 18 weeks of any primary care appointment during the study period. In total, there were 6769 patients out of 346,454 (2%) with 24,099 referrals to any treatment specialities within the 18‐week time period. Among these, 5030 patients had referrals for a minimum of one treatment speciality with possible relevance to foot and ankle health. There were 2935 patients (1%) with referrals to trauma and orthopaedics, followed by 1314 patients for paediatric services (< 1%), and 678 for physiotherapy (< 1%). For transparency, all attendances recorded in the data are presented in Supporting Information S1.

## 5 Referral Source and Attendance

**FIGURE 2:**
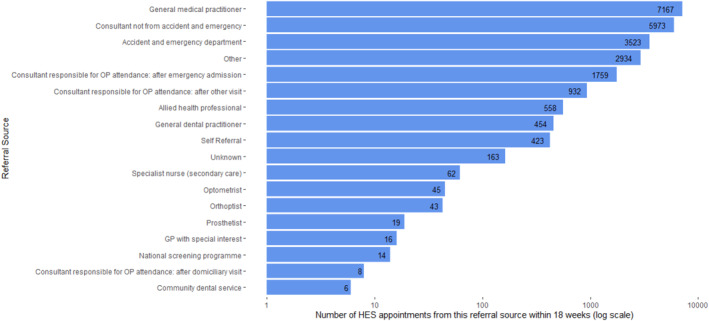
Number of Hospital Episode Statistics Outpatient appointments per referral source (*n* = 24,099) within 18 weeks of any primary care appointment from 1st January 2015 to 27th June 2020.

Figure 2 presents the 24,099 HES OP appointments according to referral source. Most were referrals from a general practitioner (7167 or 30% of appointments), followed by 5973 referrals from a consultant (other than A&E) (25%), and 3523 referrals from A&E (15%). There were 558 appointments which were referrals from allied health professionals (2%). Not all appointments were recorded as attended, there were 18,244 (76%) attended compared to 5585 (23%) not attended.

### 5.1 Diagnoses, Operations and Procedures

We used a list of ICD‐10 foot and ankle codes to search for all foot and ankle diagnoses in HES OP that were available for our linked patients. During this time period, the most frequently recorded foot and ankle diagnoses were: ‘other congenital deformities of the feet Talipes not otherwise specified’ (*n* = 291; 13%), ’other congenital malformations of the lower limb‘ (*n* = 285; 13%), ‘unspecified injury of the ankle or foot’ (*n* = 213; 10%), ‘flat foot (acquired)’ (*n* = 191; 9%) and ‘congenital deformities’ (*n* = 191; 9%).

We examined all operations and procedures in HES OP that were available for our linked patients (1st January 2015 to 31st October 2020). There were 469,298 operations and procedures among 130,902 patients. The top five code descriptions were: ‘Radiology of one body area (or < 20 min)’ (*n* = 31,039; 24%), ‘Assessment by uniprofessional team not elsewhere classifiable (NEC)’ (*n* = 23,452; 18%), ‘Assessment by multiprofessional team NEC’ (*n* = 19,560; 15%), ‘Removal of plaster cast’ (*n* = 16,632; 13%) and ‘Plain x‐ray NEC’ (*n* = 16,632; 13%).

### 5.2 Sociodemographic Variables Associated With Referrals

**FIGURE 3:**
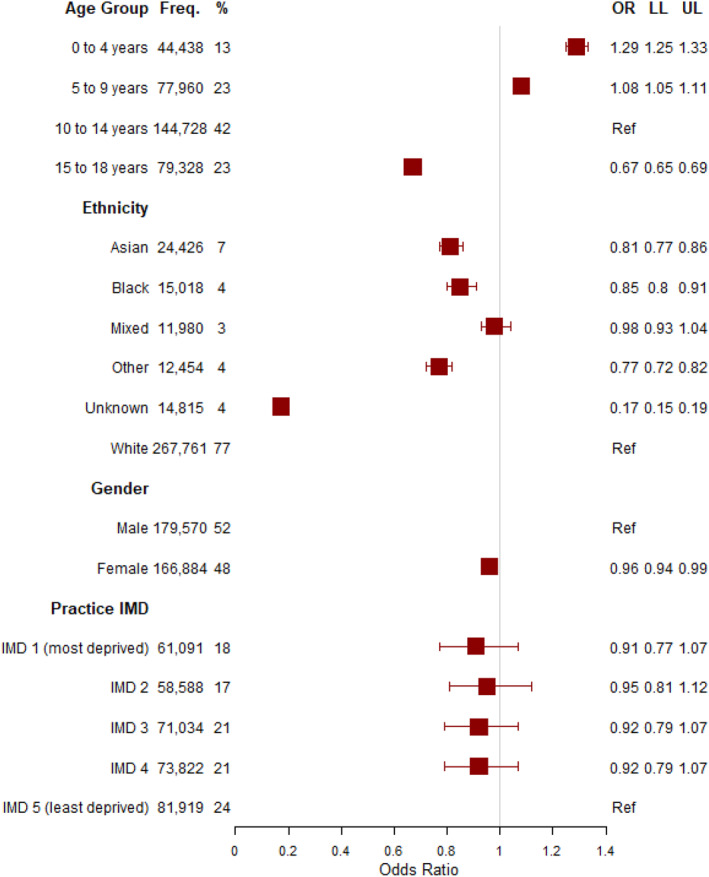
Logistic regression model of variables associated with the outcome of HES OP appointment for relevant treatment specialities within 18 weeks of any consultation for foot and ankle problems in the CPRD repeat visits for all foot and ankle encounters between 1st January 2015 and 27th July 2020. LL: lower limit; OR: odds ratio and UL: upper limit.

We examined sociodemographic associations with the outcome of HES OP appointment for relevant treatment specialities within 18 weeks of any consultation for foot and ankle problems in the CPRD for all foot and ankle encounters between 1st January 2015 and 27th July 2020 (Figure 3). Those in younger age groups had higher odds of referral compared to those aged 10–14 years, with the highest odds among those aged 0–4 years (odds ratio (OR) 1.29 and 95% confidence interval (CI) 1.25–1.33). Those aged 15–18 years had lower odds of referral compared to those aged 10–14 years (OR 0.67 and 95% CI 0.65–0.69). Those in other (OR 0.77 and 95%CI 0.72–0.82), Asian (OR 0.81 and 95%CI 0.77–0.86) and Black (OR 0.85 and 95%CI 0.8–0.91) ethnic groups had lower odds of referral compared to those in the White group. Females had slightly lower odds of referral compared to males (OR 0.96 and 95% 0.94–0.99). There were no associations observed for practice IMD compared to the reference category of the fifth quintile (least deprived), with 95% CIs for quintiles one to four including zero.

## 6 Discussion

This population‐based cohort study offers a novel analysis of linked primary and secondary healthcare data. It described the secondary care for children and young people who initially attended primary care with a foot and ankle problem in England, UK. Only 1% with foot and ankle problems had secondary care appointments recorded within 18 weeks and the specialities recorded included trauma and orthopaedics, paediatric services, physiotherapy and diagnostic imaging. The most common reasons for referral from primary care were due to musculoskeletal or unspecified pain issues and just under a third of secondary care appointments were referrals from a general practitioner. Odds for referral were higher among younger age groups compared to older (those aged 10–14 years) and children in other, Asian and Black ethnic groups had lower odds of secondary care referrals compared to those in the White group.

Foot and ankle health in children and young people is a neglected area of research, but the findings are emblematic of the large burden of morbidity in this age group driven by injuries, musculoskeletal pain and noncommunicable diseases. It is acknowledged that pathways to secondary care services are not linear, but this analysis of medical record data offers important insights into the healthcare received by children and young people. Many in our cohort had foot and ankle problems coded as musculoskeletal or “unspecified pain” but did not have a HES referral within the timeframe. In addition to musculoskeletal and unspecified pain, foot and ankle problems were also observed to be due to unintended injury, which may explain the internal referral pathways, for example, from ED to outpatient care and the high proportion of procedures for radiology, x‐ray and assessment. These data provide insight into the delivery of healthcare services and can be used to inform discussion about service planning and allocation of resources. We have captured the breadth of health professionals in secondary care involved in the care of children with foot and ankle problems and these include Allied Health Professionals (AHPs), including physiotherapists and podiatrists, who can generate beneficial outcomes for children and young people with foot and ankle problems [15]. Further analysis of routine data from community services is warranted to understand the different pathways between primary care and specialist services. These data are critical to informing the delivery and evaluation of interventions and further debate about role and service expansion.

Analysis of linked primary care, outpatient and emergency department (ED) visits in England has been used to estimate health service use in infants, children and young people [6]. Following analysis of data from 2007 to 2017, a downward trend in primary care consultation rates for all children except infants was reported [6], along with an increase in use of ED and outpatient care in all aged under 15 years [6]. Although there are multiple factors that contribute to these findings, the authors identified that difficulties with accessing primary care and limited community services may be one of the drivers into outpatient and ED care. There is increasing use of emergency departments for management of musculoskeletal conditions [26] and ongoing debate about the core competencies of health professionals seeing children and young people [26, 27, 28]. Through understanding the breadth of conditions presenting into secondary care services, these data could help to define priorities for training for healthcare professionals working in these services.

One of the most important findings from our analysis was that children and young people belonging to other, Asian and Black ethnic groups had lower odds of referral to secondary services. This finding echoes our previous work [2], where children and young people from ethnic minority groups had lower odds of repeat consultations. Although there is limited evidence documenting the factors specific to repeat attendance for foot and/or ankle problems, our findings align with a recent scoping review which identified inequalities with healthcare utilisation [29] amongst children from different ethnic groups. This work primarily focused on primary care and acknowledged that emergency and outpatient care was poorly studied as was the breadth of health conditions reported in their review. The factors underpinning inequalities are complex [30] and, given the limitations with the completeness of the data used in this study, some caution must be drawn. Nevertheless, more work to understand the barriers to healthcare for children and young people from ethnic minority groups should be a priority.

The first step towards transformative research and improved health outcomes is quality data and mandatory diagnostic coding in outpatient care and linked community care data. CPRD Aurum is a large database with established representativeness, validity and quality [16]. HES OP also has established validity [17], but our analysis highlights its limitations. There was a high proportion of generic or unknown specialities and referral sources. We made the decision to assume patients with referrals to generic specialities, such as ‘paediatric services‘, were relevant to foot and ankle health, which may have led to the over‐estimation of the outcome at analysis stage. Ultimately, we cannot determine whether children and young people attended other specialities or vice versa—that children and young people with concerns not relevant to foot and ankle health were included as attending a so‐called relevant speciality. Ideally, we would have utilised diagnostic coding to establish reasons for attendance; however, such coding is not mandatory for outpatient care, restricting our understanding of how patient needs are being met in this part of the health service. There are alternative HES datasets where diagnostic coding is more complete, but these have limited applicability to foot and ankle health.

## 7 Conclusion

This is one of the largest studies describing the secondary care services for children and young people with foot and ankle problems. Only a small proportion of those with foot and ankle problems presenting in primary care also had HES OP referral recorded within 18 weeks and the most common speciality accessed was trauma and orthopaedics. Sociodemographic differences in secondary care referrals were identified and could be indicative of inequalities with access to services and should be a priority for further research. This analysis of linked primary and secondary population‐level data has enhanced our understanding in this under‐researched field, but the lack of mandatory diagnostic coding in outpatient care is a key limitation.

## Author Contributions

**Emma Rezel‐Potts:** conceptualization, methodology, project administration, data curation (lead), software (lead), formal analysis (lead), writing – original draft, writing – review and editing. **Catherine Bowen:** funding acquisition, conceptualization, methodology, writing – original draft, writing – review and editing. **Kate M. Dunn:** funding acquisition, conceptualization, methodology, writing – original draft, writing – review and editing. **Christopher I. Jones:** funding acquisition, conceptualization, methodology, data curation, formal analysis, writing – original draft, writing – review and editing. **Martin C. Gulliford:** methodology, data curation, supervision, formal analysis, writing – original draft, writing – review and editing **Stewart C. Morrison:** funding acquisition, conceptualization, methodology, supervision, writing – original draft, writing – review and editing.

## Ethics Statement

The study protocol was reviewed via Research Data Governance Application and approved by the CPRD team (protocol number 20_ 002,137).

## Consent

This was an analysis of medical record data and consent was not required for the purposes of this analysis. However, CPRD has an ethical approval from the Health Research Authority to support research using anonymised patient data.

## Conflicts of Interest

Dr Stewart Morrison is the deputy editor of the Journal of Foot and Ankle Research. All other authors declare no conflicts of interest.

## Supporting information

Figure S1

## Data Availability

The study is based on data from the Clinical Practice Research Datalink (CPRD) obtained under license from the UK Medicines and Healthcare Products Regulatory Agency (MHRA); however, the interpretation and conclusions contained in this report are those of the authors alone. All proposals requesting data access will require approval from CPRD.
