# Supplementary material for: Primary Care and Linked Secondary Care Encounters for Foot and Ankle Problems in Children and Young People: A Population‐Based Cohort Study in England
Source: J Foot Ankle Res. 2025 Apr 15;18(2):e70046. doi: 10.1002/jfa2.70046 (PMC11999890; doi:10.1002/jfa2.70046)
Supplement: Supplementary file 1 — Figure S1 [file JFA2-18-e70046-s001.docx]

Supplementary File 1

**
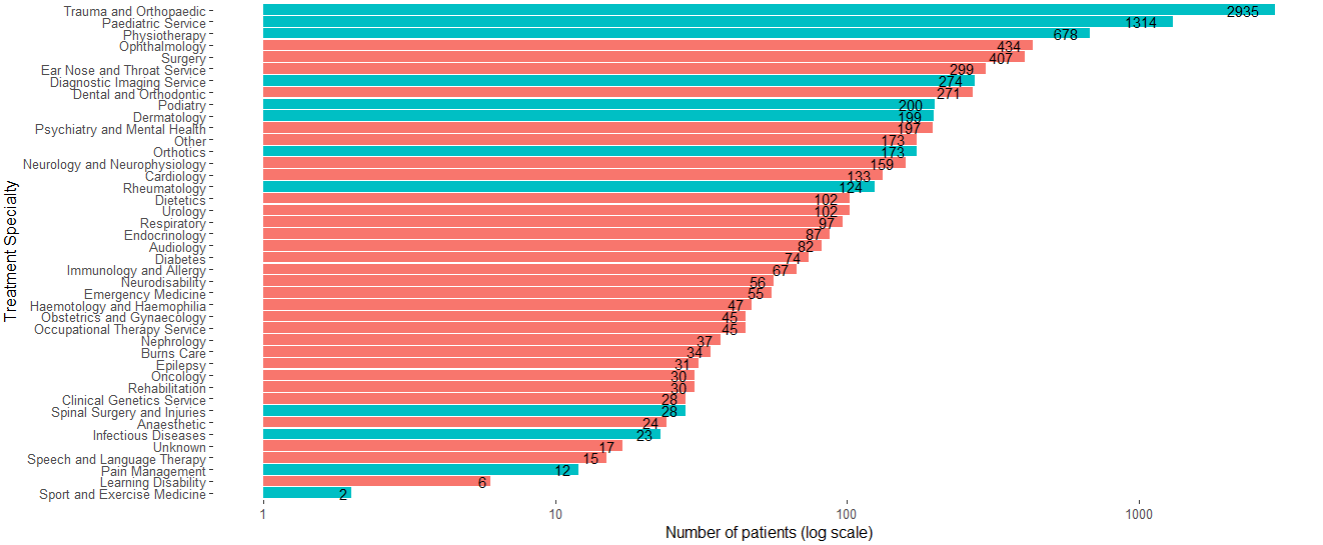
**Figure S1 Number of patients referred to Treatment Specialities in Hospital Episode Statistics Outpatient Records within 18 weeks of any primary care appointment from 1^st^ January 2015 to 27^th^ June 2020. Individual patients may have referrals for more than one specialty. Blue bars indicate relevant categories of treatment specialty.
